# Prevalence of Pseudobulbar Affect (PBA) in Parkinson’s Disease: An Underrecognized Patient Burden

**DOI:** 10.7759/cureus.19960

**Published:** 2021-11-28

**Authors:** Ramsey Falconer, David Whitney, Hannah Walters, Sean Rogers

**Affiliations:** 1 Neurology, Inova Parkinson's and Movement Disorders Center, Falls Church, USA

**Keywords:** parkinson's disease, pseudobulbar affect, pba, emotional lability, depression, cns-ls

## Abstract

Objective

Pseudobulbar affect (PBA) is a neurological condition characterized by emotional lability and a discrepancy between the patient’s emotional expression and emotional experience. These uncontrollable episodes cause distress in social situations resulting in embarrassment and social withdrawal. The most comprehensive study to date estimated that 26% of Parkinson’s disease (PD) patients screened positive for PBA symptoms via the validated Center for Neurologic Study-Lability Scale (CNS-LS) screening tool. We hypothesize that the prevalence of this disabling syndrome is higher than reported, often being labeled as depression.

Methods

One hundred patients were enrolled in the study and screened with a CNS-LS tool, all of whom were diagnosed with PD by a fellowship-trained movement disorder specialist. Patients were also asked about previous diagnosis of depression, current antidepressant medication use, and history of PBA diagnosis and treatment.

Results

The percentage of PD patients (n = 100) with PBA symptoms as defined by a CNS-LS score ≥13 was 41% (n = 41) and by a CNS-LS score ≥17 was 21.0% (n = 21). In our sample, 38.0% of patients (n = 38) had a previous clinical diagnosis of depression and 25.0% (n = 25) were currently undergoing treatment for their depression. There was a significant association between previous depression diagnosis, current antidepressant use, and higher CNS-LS scores (p < 0.001).

Conclusion

Using either of the CNS-LS score cutoffs, a significant proportion of the PD population in our sample displayed symptoms of PBA. We also found an association between previous diagnosis of depression and higher CNS-LS scores as well as between antidepressant use and higher CNS-LS scores. This suggests both a higher prevalence than prior studies showed as well as frequent misdiagnosis or co-diagnosis with depression.

## Introduction

In treating Parkinson’s disease, symptoms of depression, anxiety, and emotional lability are frequently brought up at clinical visits. This presents in many ways: depressed mood, abulia or other lack of motivation, fluctuating anxiety, or a generalized sense of unwellness. Depression is found in 30-40% of patients diagnosed with Parkinson’s disease, but only 20% or less receive treatment [1].

Symptoms such as these are at times difficult to elicit in a clinical visit, be it due to time constraint and more pressing clinical symptoms which take precedence or even reticence of patients to bring up mood-based symptoms. Regardless, the nonmotor symptoms of depression can exert an impact on a patient’s daily functioning and wellbeing.

Complicating a diagnosis of depression is the potential diagnosis of pseudobulbar affect (PBA), which in many ways mimics a diagnosis of depression, but in many ways, is very different. PBA is a neurological condition characterized by emotional lability and highlighted by a discrepancy between the patient’s emotional expression and their emotional experience. It commonly manifests as involuntary, sudden, and recurrent episodes of laughing and/or crying that tend to be inappropriate or disproportionate to the social context or stimuli [2]. This is different from depression, where the emotional response tends to be to a degree in line with their emotional state or current environmental stimuli. The uncontrollable episodes of PBA cause considerable distress in social situations resulting in embarrassment and social withdrawal [2]. PBA is often confused with mood disorders, but there is a key difference between both diagnoses. Mood disorders, such as depression, represent one’s emotional state over an extended period, whereas the emotional state seen in PBA is more labile with varying episodes of explosive and incongruent behavior due to a lack of ability to regulate emotional expression [3].

PBA occurs secondary to many neurological conditions or injury, including Parkinson’s disease (PD), amyotrophic lateral sclerosis (ALS), multiple sclerosis (MS), stroke, traumatic brain injury (TBI), and Alzheimer’s disease (AD) [4]. The pathophysiology of PBA, however, is complex and not fully understood. The most accepted hypothesis is that PBA occurs due to dysfunction of the cortico-pontine-cerebellar circuit and involves the motor cortex. Since the cerebellum normally controls emotional expression based on information received from the motor cortex, a disruption in this circuit leads to PBA [5]. This could explain the increased prevalence of PBA in atypical parkinsonian disorders, including progressive supranuclear palsy (PSP) and the cerebellar subtype of multiple system atrophy (MSA-C), both of which affect brain structures involved in regulating emotional expression [3]. That being said, PBA is a syndrome associated with nearly every form of neurological dysfunction, including Parkinson’s disease [4].

With an estimated United States prevalence of 1.8 - 7.1 million individuals, PBA is believed to be under-recognized and undertreated, especially in the parkinsonian population [6]. The prevalence of PBA in other neurologic conditions seems to be much higher, with estimates of 12-70% in ALS, 10-50% in MS, 5-60% in stroke, 5-80% in TBI, and 9-40% in AD, as compared to 3.6-42.5% in Parkinson’s disease patients [3]. Other studies have shown that the prevalence of PBA increases with the disease progression of PD, even as apathy and abulia increase [7].

While some screening tools have been validated for PBA in other neurological conditions such as MS and ALS, there is limited research addressing PBA in the PD population [8,9]. In this study, a validated screening instrument for PBA in MS and ALS, the Center for Neurologic Study-Lability Scale (CNS-LS) [2], was applied to explore the prevalence of PBA symptoms in Parkinson’s Disease. This screening tool was validated in a large clinical trial and is considered the standard screening tool used clinically to help identify PBA. We report the patient data and CNS-LS scores collected in our institution.

## Materials and methods

All patients who presented to the movement disorder clinicians of our institution over a two-week period were enrolled in the study. The study was considered a quality improvement project by the local IRB, and an IRB waiver was issued. A total of one hundred patients were included. Inclusion criteria were formal diagnosis of PD by a fellowship-trained movement disorder specialist and ability to complete the CNS-LS. Exclusion criteria included the inability to take the CNS-LS as well as prior diagnosis of PBA. The CNS-LS is a self-reported measure of PBA consisting of three questions intended to screen for symptoms of inappropriate crying and four questions directed at symptoms of inappropriate laughter. Each question is rated on a Likert scale of one (applies never) to five (applies most of the time), with a possible overall score ranging from seven to 35 [10]. The CNS-LS has been validated for PBA screening in MS and ALS. CNS-LS scores of ≥13 have been demonstrated as reliable cutoff scores to suggest a diagnosis of PBA in patients with MS (72%), and a cutoff of ≥17 was shown to be even more reliable of a cutoff score to suggest a diagnosis of PBA in patients with MS (89%) [8,9]. In this study, CNS-LS scores of ≥13 and ≥17 were assessed. Patients were also asked about previous diagnosis of depression, current antidepressant medication use, and history of PBA diagnosis and treatment.

## Results

**Figure 1:**
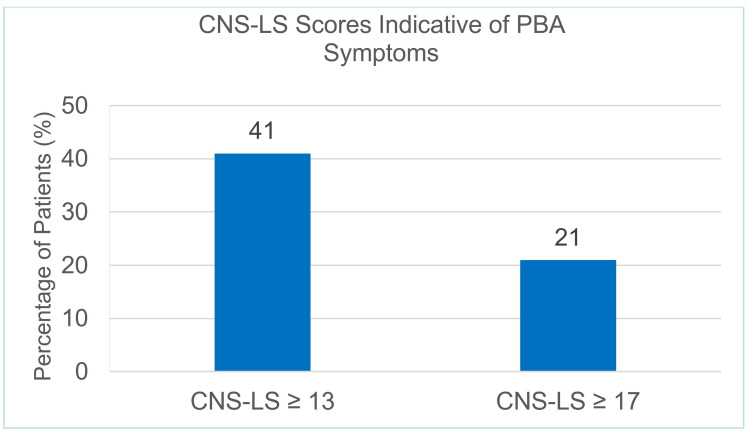
Percentage of patients with CNS-LS scores of ≥13 and ≥17

In our sample, 41% (n = 41) of patients screened positive for PBA using CNS-LS score ≥ 13, and 21.0% (n = 21) using CNS-LS score ≥ 17 (Figure 1). The average CNS-LS score reported was 12.0, with a standard deviation of 4.7.

Demographic characteristics and psychiatric variables that were also collected are reported in Table 1. The mean patient age was 72.1 years with a standard deviation of 8.7 years. The majority of our patients were male (n = 59, 59.0%).

**Table 1:** Patient data PBA - pseudobulbar affect, CNS-LS - Center for Neurologic Study-Lability Scale

Variable	Mean (SD) or percentage
Demographic characteristics (n = 100)	
Age in years	71.8 (8.7)
Sex	
Male (n = 59)	59.0%
Female (n = 41)	41.0%
Psychiatric variables	
Previous depression diagnosis (n = 38)	38.0%
Current antidepressant use (n = 25)	25.0%
PBA screening tool	
CNS-LS score (n = 100)	12.2 (4.8)

Looking further into the co-diagnosis and treatment of depression, a logistic regression analysis was also conducted, with the result showing a strong association (p<0.0001) between CNS-LS scores and depression diagnosis or taking an antidepressant. A considerable number of patients in our sample had a previous clinical diagnosis of depression 38.0% (n = 38), and 25.0% (n = 25) were currently undergoing treatment for their depression (Table 2). There were consistent and strong associations with both of these psychiatric variables and higher CNS-LS score.

**Table 2:** CNS-LS scores and association with depression and treatment of depression CNS-LS - Center for Neurologic Study-Lability Scale

	Overall	CNS-LS Score <13	CNS-LS score ≥ 13	OR (95% confidence interval)
Age	71.9 ± 8.8 (100)	72.6 ± 9.5 (59)	70.8 ± 7.6 (41	0.976 (0.932-1.022)
Gender				
Female	41 (41.00%)	23 (56.10%)	18 (43.90%)	Referent
Male	59 (59.00%)	36 (61.02%)	23 (38.98%)	0.816 (0.364 – 1.833)
Diagnosed depression				
No	62 (62.00%)	46 (74.19%)	16 (25.81%)	Referent
Yes	38 (38.00%)	13 (34.21%)	25 (65.79%)	5.529 (2.295 – 13.317)
Currently undergoing depression treatment				
No	75 (75.00%)	52 (69.33%)	23 (30.67%)	Referent
Yes	25 (25.00%)	7 (28.00%)	18 (72.00%)	5.812 (2.135 – 15.823)

## Discussion

Depression, or symptoms that we naturally associate with depression, are common in the Parkinson’s disease population [1], and when present, can influence many of the other systemic symptoms of Parkinson’s disease. The presence of depressed mood, abulia or other lack of motivation, fluctuating anxiety, or a generalized sense of unwellness can impact the patient’s subjective daily experience through many ways. This includes everything from a potential impact on sleep, motivation to exercise as well as playing a part in daily choices on social and emotionally stimulating activities [11].

This leads in many clinical settings to the postponement of topics of depression, mood changes, and more as a patient’s list of problems tend to be more visibly present (tremor, gait changes, falls) instead of emotional, as a stigma still exists related to mental illness both socially and within healthcare [12]. By doing so, an opportunity is missed to discuss common symptoms that negatively impact a patient’s quality of life measures and leads to degradation of a patient’s social and emotional foundation [13,14].

Complicating the picture is the potential presence of PBA, a syndrome unique unto itself and easily labeled as depression/anxiety, as many of the symptoms clinically appear to overlap. But PBA is a clinically distinct syndrome with a specific diagnostic criterion, as well as a unique disease-specific treatment usually identified via CNS-LS screening and clinical history [5]. 

Using either of the CNS-LS score cutoffs, the data show that a large percentage of the PD population in our sample display symptoms of PBA (41% using ≥ 13 and 21% using ≥ 17). These are far higher than were reported in the PRISM study, which found only 26% of patients had a CNS-LS score of at least 13 [4]. They are also much higher than those of another study by Strowd et al., which found only 23.8% and 7.1% of PD patients had a CNS-LS score of at least 13 and 17, respectively [10]. The study by Strowd et al. was limiting, though, as the team used only three items of the CNS-LS and added four of their own, not the seven-question scale in the current CNS-LS. Regardless, our data suggest that the prevalence of PBA is possibly higher in the PD population than previously considered in either of the prior studies, and the overlap with a diagnosis of depression also appears to be common.

As evidence of this, a large percentage of the PD population in our sample have a previous diagnosis of depression (36.6%), and many are currently using antidepressant medication (23.7%). Furthermore, a statistically significant association exists between a previous diagnosis of depression and higher CNS-LS scores (p < 0.001), as well as antidepressant use and higher CNS-LS scores (p < 0.001). This may be in part due to the symptomatic similarities of PBA and depression, these symptoms being diagnosed and treated as depression, or the diagnosis of depression masking a diagnosis of PBA in a clinical setting by a label of depression. 

This reflects the complicated nature of a screening tool such as the CNS-LS, which is designed to capture positive responses with a risk of false-positive results. Depression and the symptoms of depression could also be present, as co-diagnoses of PBA and depression are possible. This could skew CNS-LS positivity rates higher, especially in a disease state such as Parkinson’s, where depression is so common [2].

This suggests the need for further investigation into the validity of the CNS-LS within the Parkinson’s disease population given the marked variability of estimates of the prevalence of PBA in PD patients across multiple studies, including this one, which could be in part due to the potential for false positives. One study by Phuong et al. found that when using formal diagnostic interviews to diagnose PD patients with PBA, 7% met criteria for PBA, whereas 42.5% met criteria using the CNS-LS threshold of 13 [15]. This highlights the disparity between different methods of diagnosing PBA, and if anything, further emphasizes the need for a standardized measure to diagnose PBA in the PD population. While the CNS-LS has been validated for other neurological conditions such as MS and ALS, it has not yet been validated for use in PD.

## Conclusions

The data from this study and the context of this data illustrates the role of the CNS-LS as a screening tool not to in-and-of-itself give a diagnosis but to be used as the launching point for a conversation about emotionality and mood. This then could potentially then lead to diagnosis and treatment, be the root cause of PBA, depression/anxiety, or a mixture of both. If, as this data suggests, nearly 40% of the PD patient population is scoring a CNS-LS score high enough to suggest the possibility of PBA, further education on the pathology and neuropsychological burden of PBA, as well as depression, is warranted to expand the conversation on these socially isolating and emotionally burdensome conditions. And if in the process, we can begin to unravel the stigma around discussing emotional and mental health concerns to lead to a more honest conversation with our patients on the topic of depression, anxiety, and PBA, then that counts as a win.
